# High-Performance Multifunctional Photodetector and THz Modulator Based on Graphene/TiO_2_/p-Si Heterojunction

**DOI:** 10.1186/s11671-021-03589-w

**Published:** 2021-08-21

**Authors:** Miaoqing Wei, Dainan Zhang, Lei Zhang, Lichuan Jin, Huaiwu Zhang

**Affiliations:** 1 School of Electronic Science and Engineering (National Exemplary School of Microelectronics), University of Electronic Science and Technology of China, Chengdu, 611731 China; 2 State Key Laboratory of Electronic Thin Films and Integrated Devices, University of Electronic Science and Technology of China, Chengdu, 611731 China

**Keywords:** Multifunctional device, Graphene/TiO_2_/p-Si, Photodetector, Broadband THz wave modulator

## Abstract

**Abstract:**

In this paper, we have reported a multifunctional device from graphene/TiO_2_/p-Si heterojunction, followed by its systematical analysis of optical response in a device under ultraviolet–visible-infrared band and transmission changes of terahertz waves in the 0.3–1.0 THz band under different bias voltages. It is found that photodetector in the “back-to-back” p-n-p energy band structure has a seriously unbalanced distribution of photogenerated carriers in the vertical direction when light is irradiated from the graphene side. So this ensures a higher optical gain of the device in the form of up to 3.6 A/W responsivities and 4 × 10^13^ Jones detectability under 750 nm laser irradiation. Besides, the addition of TiO_2_ layer in this terahertz modulator continuously widens the carrier depletion region under negative bias, thereby realizing modulation of the terahertz wave, making the modulation depth up to 23% under − 15 V bias. However, almost no change is observed in the transmission of terahertz wave when a positive bias is applied. A similar of an electronic semiconductor diode is observed that only allows the passage of terahertz wave for negative bias and blocks the positive ones.

**Graphic Abstract:**

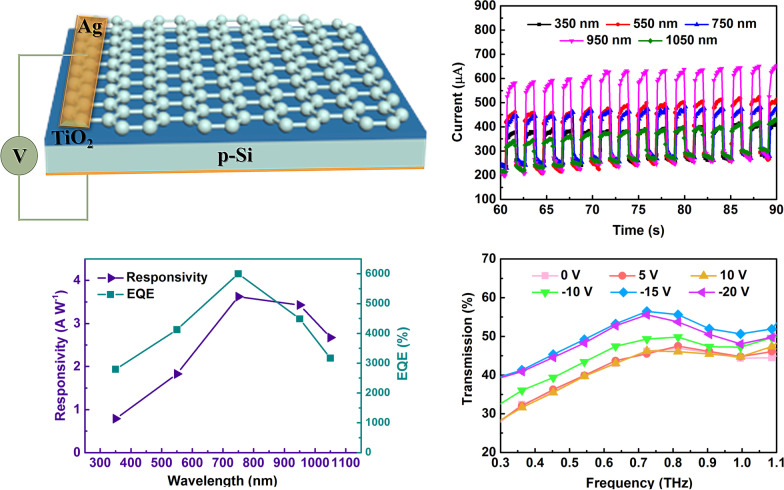

## Introduction

An optoelectronic device such as a photodetector converts the light signal into an electrical signal and is widely used in optical communications, thermal imaging, environmental monitoring, biomedical imaging, etc. [1–5]. Terahertz (THz) broadband modulator is a key device to realize the practical application of terahertz wave in communication, imaging, sensing and other application areas [6–10]. Therefore, the development of a high-speed modulator that works at room temperature, small in size, and easy to process, is of great significance for the development and practicality of terahertz technology.

Recently, graphene has been employed in the field of photodetectors and terahertz modulators due to its special energy band structure and ultra-high carrier mobility [11–16]. So, graphene-based multifunctional photodetectors and terahertz modulators can overcome the above issue due to its miniaturization of electronic communication systems. Graphene-based photodetectors have been extensively studied in the past few years. Graphene/Si heterojunction has high photoelectric conversion efficiency and thus has great potential to obtain high-performance photodetectors [17–20]. However, the low optical absorption of graphene (~ 2.3% for a single layer of graphene) is responsible for low responsivity [21, 22]. The interface between Si and graphene contains a large number of surface states pinning the surface Fermi level, leading to a strong leakage current noise. Various methods have been proposed to improve the performance of graphene/silicon photodetectors, including insertion of an interfacial oxide layer, silicon waveguide integration, optical absorption layer addition, and surface plasmon enhancement [23–29].

In the case of graphene-based terahertz modulators, both optical pump and external voltage bias can be used as excitation signals. To perform all-optical modulation of terahertz waves, Wen et al. [30] proposed a modulator where a layer of graphene is placed on a germanium substrate. When the sample is irradiated with a 1550 nm communication wavelength laser, the terahertz transmission intensity sharply dropped along with the increase in laser power. The modulation depth reaches 94% in the frequency range of 0.25–1 THz. Professor Sensale et al. [31] used a graphene-based field effect transistor (GFET), where 92 nm SiO_2_ is used as a dielectric layer of terahertz modulator with a modulation depth of 15% and modulation rate of 18 Kb/s. This type of THz wave wide-band modulator completely uses electron and hole concentration changes in the graphene film for the control attenuation of terahertz wave energy. After that, aluminum oxide Al_2_O_3_, YIG, and other materials [32–34] can effectively reduce the Coulomb scattering and cavity effect of graphene due to their high dielectric constant and thus are used as the dielectric layer of GFET terahertz wave modulator.

In this study, graphene/TiO_2_/p-Si trilayer heterojunction is fabricated to design a photodetector and a broadband THz wave modulator. Moreover, our designed device can also work as a diode for terahertz waves that can only pass the current of negative bias and blocks the positive one.

## Methods

**Fig. 1:**
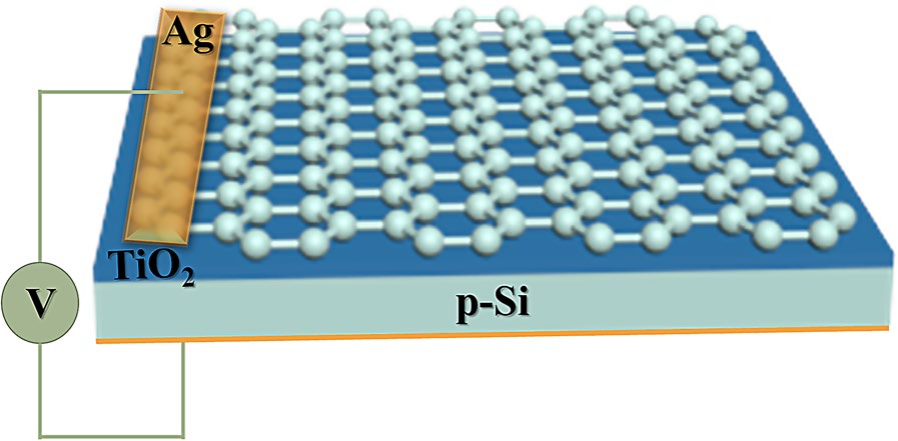
Schematic of graphene/TiO_2_/p-Si heterojunction-based multifunctional device

The prototype of a multifunctional photodetector and THz modulator based on graphene/TiO_2_/p-Si heterojunction is shown in Fig. 1. The TiO_2_ film is deposited on the p-Si substrate (500-μm-thick, resistivity *ρ* ~ 1–10 Ω cm), using the low-temperature hydrothermal method. The cleaned silicon substrate was immersed into 0.1 M TiCl_4_ aqueous solution at 343 K for one hour to obtain about 10-nm-thick TiO_2_ film. A single layer of graphene is grown on the copper substrate by chemical vapor deposition [35], then spin-coated with PMMA, and immersed into 1 mol/L FeCl_3_ solution for 90 min to remove the copper background. After that, the TiO_2_/p-Si substrate was used to pick up the PMMA-coated monolayer graphene and then dry it to remove PMMA [36], and get graphene/TiO_2_/p-Si trilayer heterojunction, the PMMA-coated graphene/TiO_2_/p-Si substrare is immersed in acetone solution. The photoresponses of graphene/TiO_2_/p-Si heterojunction, such as photogenerated current and time-dependent characteristics, were measured on a monochromator (Zolix, Omni-λ 300i), which use order sorting filters to provide white and monochromatic light. The *I*–*V* curves and *I*–*t* curves of the graphene/TiO_2_/p-Si heterojunction under light illumination were measured by a SourceMeter (Keithley 2601B). The terahertz wave transmission was measured by a Fico THz time domain system (Zomega Terahertz Corporation).

## Results and discussion

**Fig. 2:**
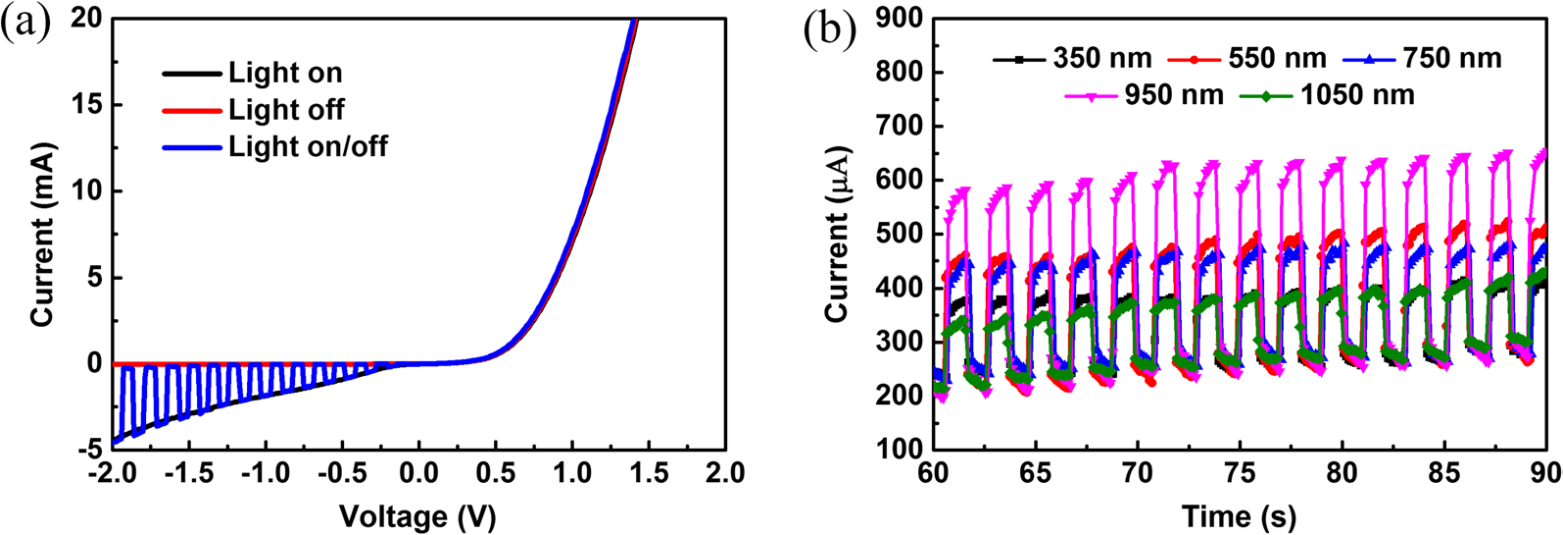
The photoresponse measurements. **a** IV and time-dependent IV curves of graphene/TiO_2_/p-Si heterojunction. **b** The curves of graphene/TiO_2_/p-Si heterojunction at various wavelengths of light

The dark current of the device and the photocurrent when excited by white light changes with the bias voltage, and change in current with a voltage of 2 Hz white light is shown in Fig. 2a. From Fig. 2a, we can see that the device has an obvious rectifying effect. The positive direction of applied voltage is from the silicon to graphene side, which indicates that the main built-in electric field of the device is due to silicon-titanium oxide junction. On the other hand, the built-in electric field introduced by weak p-type graphene [37, 38] and weak n-type TiO_2_ can be ignored. Under the negative bias voltage, the built-in electric field is further enhanced by the external bias voltage. When light is irradiated from the graphene side, a large number of photogenerated carriers are collected by the graphene which is quickly transmitted out. This results in an imbalance between the photo-generated carriers on the graphene and silicon side, which consequences in a strong optical guide gain under high bias. When 2 Hz white light is applied, the current coincides with the dark current (no light) and coincides with the photocurrent (when light is applied), which shows that the device has a high response speed and good repeatability. As shown in Fig. 2b, graphene/TiO_2_/p-Si has an obvious photoelectric response in the range of 350–1050 nm wavelength at a bias of − 2 V. Furthermore, it can be seen from Fig. 2 that the dark current of the device does not significantly change under different wavelengths of laser irradiation. However, the dark current slightly increases along with the increase in time. This is caused by the heat loss when the device is working. When the working time of the device is extended, a local temperature is raised, which causes an increase in leakage of current. Photocurrent of the device obviously changes under different light waves (350–1050 nm). The photoresponse currents (*I*_on_ − *I*_off_) at 350, 55, 750, 950, and 1050 nm are 126.29, 213.35, 189.67, 335.93, and 102.46 μA, respectively.

**Fig. 3:**
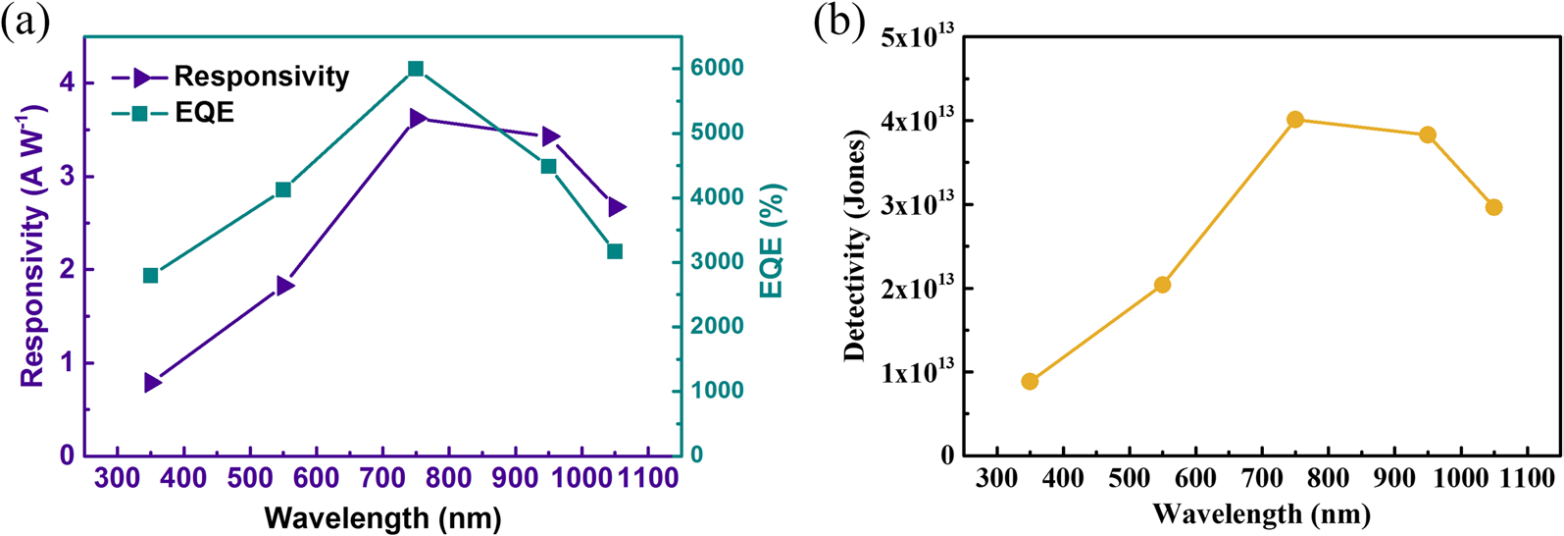
Performance of graphene/TiO_2_/p-Si heterojunction. **a** The spectra-dependent responsivity and external quantum efficiency of graphene/TiO_2_/p-Si heterojunction. **b** The spectra-dependent detectivity of graphene/TiO_2_/p-Si heterojunction

The spectra-dependent of responsivity (*R*), external quantum efficiency (EQE), and detectivity (D*) of the heterojunction are shown in Fig. 3a, b. The performance of photodetectors can be evaluated from the *R*, EQE and D* parameters. Responsivity (*R*) is defined as *R* = (*I*_on_ − *I*_off_)/*P*_in_, where *I*_on_, *I*_off_ and *P*_in_ are photocurrents, dark current, and incident light power. External quantum efficiency (EQE) is the number of electron–hole pairs excited by the unit incident photon, which reflects the sensitivity of photodetectors to photons and can be calculated as EQE = *Rhc*/*λ*. Here *h*, *c* and *λ* are Plank’s constant, elementary charge, and wavelength of incident light, respectively. Detectivity (D*) can be expressed as D* = *R*/(2*qI*_d_/*A*)^1/2^, where *I*_d_ is the dark current and *A* is an active area (0.5 cm^2^). The highest responsivity and EQE of 3.6 A/W and 6001% are observed under light with 0.417 mW/cm^2^ intensity and 750 nm wavelength. Detectivity is 4 × 10^13^ Jones at 750 nm light, which is about 520 times higher than the graphene/Si photodetector [39]. Light is irradiated from the graphene side, and the carriers excited by the short-wavelength are near the TiO_2_ side. At this time, the electrons are quickly collected by the graphene under the negative bias voltage. However, holes could not travel a long distance on the silicon side, cause charge recombination, and reduce the photoresponse of the device. Besides, insufficient absorption of long-wavelength light by Si can also reduce the photoresponse of the device. It is found that excitation light of about 750 nm wavelength can be completely absorbed by Si, and the distribution of photogenerated carriers in the thickness direction of the device is relatively uniform, so the best responsivity is obtained.

**Fig. 4:**
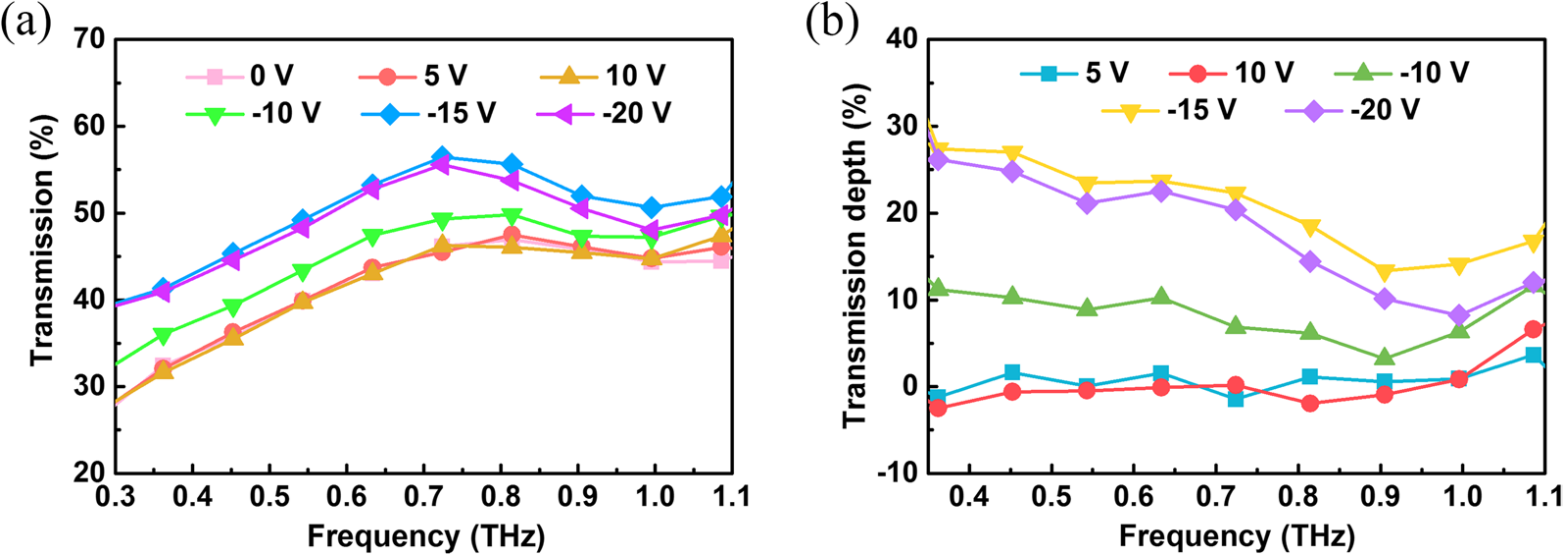
Modulation test. **a** Transmittance spectra of graphene/TiO_2_/p-Si at various gate bias voltages. **b** Transmission modulation depth as functions of voltage for graphene/TiO_2_/p-Si heterojunction

The THz wave transmittance of THz wave of the graphene/TiO_2_/p-Si heterojunction in the range of 0.3–1 THz is shown in Fig. 4a. It can be seen from Fig. 4a that when a positive bias voltage of 5 V and 10 V is applied, the transmittance of the THz wave hardly changes compared to 0 V. When negative bias voltages of − 10 V, − 15 V, and − 20 V are applied, the THz wave transmittance significantly changed. The direction of the applied negative bias electric field is the same as the direction of a built-in electric field of p-Si and TiO_2_. As the negative bias voltage increases, the space charge region widens, and the device gradually becomes fully depleted. Meanwhile, there is no carrier accumulation inside the device, the carriers move along the external circuit, and transmission of the terahertz wave increases. The modulation depth is an important performance parameter of terahertz modulators, which can be calculated by (*T*_excitation_ − *T*_no excitation_)/*T*_no excitation_, where the *T*_excitation_ and *T*_no excitation_ represent the intensity of THz transmission with and without photoexcitation, respectively. The variation in modulation depth of the device under different bias voltages in the range of 0.3–1.0 THz is calculated, and the result is shown in Fig. 4b. It can be seen from Fig. 4b that when 5 V and 10 V are applied, the modulation depth is approximately zero. The modulation depth is about 23% at − 15 V. While at − 20 V, the modulation depth slightly decreased to about 22.6%. The reason behind this is the use of extremely high voltage, where the device breaks down in the reverse region and the current increases. Continuing to increase the voltage will not further broaden the space charge layer to increase THz transmission, but will increase the temperature of the device and increase the carrier concentration due to the thermal effect, caused by the increase in current. This decreases terahertz wave and resulting in the decrease of modulation depth.

**Fig. 5:**
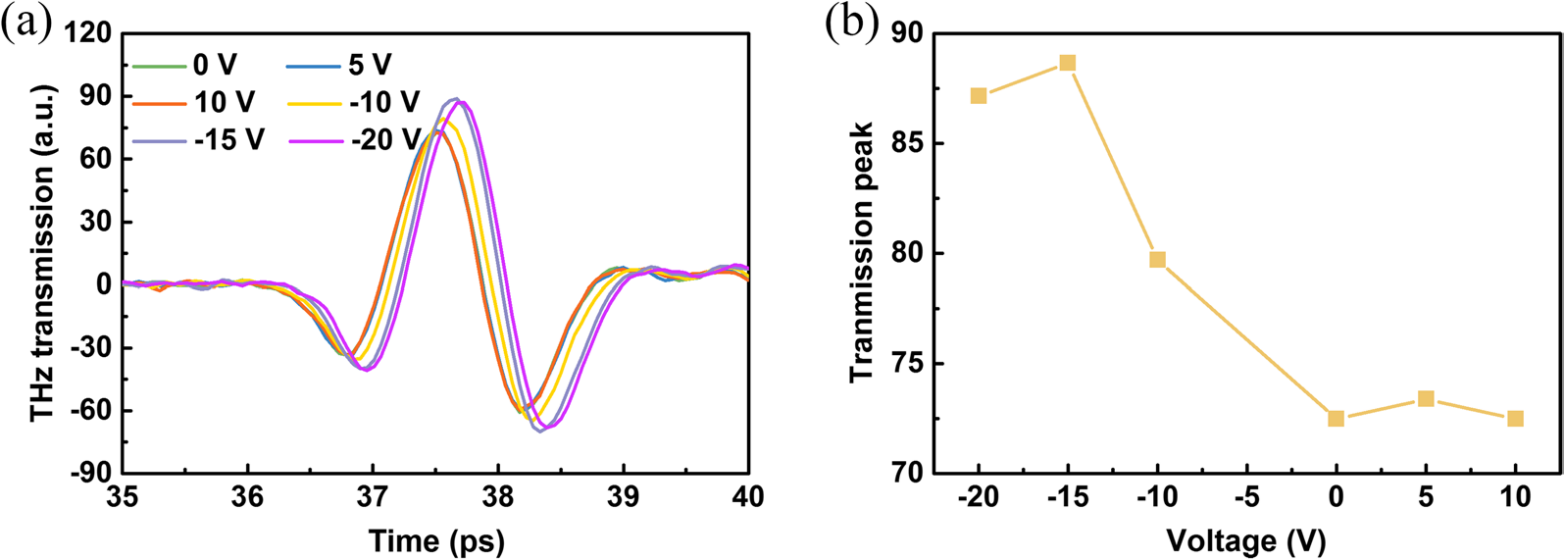
The terahertz time-domain signals. **a** The terahertz time-domain signals at various gate bias voltages. **b** Gate voltage-dependent time-domain terahertz transmission peaks of graphene/TiO_2_/p-Si

Time-domain signals of graphene/TiO_2_/p-Si THz modulator with various bias voltages are plotted in Fig. 5a. We can see from the time-domain graph when a positive gate voltage is applied almost coincides with the graph when 0 V is applied. When a negative bias is applied, the peak of THz transmission significantly increases. The peak value at 0, 5, 10, − 10, − 15, and − 20 V is about 72.49, 73.39, 72.49, 79.7, 88.66, and 87.15, respectively. In order to clearly show this change, we plotted the change of device's terahertz transmission peak under different voltages in Fig. 5b. It is clear that the device only allows terahertz waves to pass under negative bias, but prevents terahertz waves from passing under positive bias. So, it is inferred that our terahertz modulator can also function as a diode for terahertz waves.

## Conclusions

In summary, it is reported that graphene/TiO_2_/p-Si heterojunction can be used as a high-performance photodetector and a broadband THz wave modulator. Our proposed device has a distinct light response in the ultraviolet–visible-infrared band. The responsivity, external quantum efficiency, and detectivity are as high as 3.6 A/W, 6001%, and 4 × 10^13^ Jones, under 0.417 mW/cm^2^ density and 750 nm wavelength laser irradiation. Moreover, the transmission modulation depth of terahertz wave reaches up to 23% in the broadband of 0.3–1.0 THz at a biased voltage of − 15 V, while no any obvious change is observed in the transmission of the terahertz wave under a positive voltage, which concludes that this device can function as “a diode” relative to the terahertz wave.

## Data Availability

All data supporting the conclusions of this article are included within the article.

## Abbreviations

THz: Terahertz
GFET: Graphene-based field effect transistor
R: Responsivity
EQE: External quantum efficiency
D*: Detectivity
